# Psychological Distress as a Predictor of Subsequent Loneliness Among Japanese University Students: A Cross-Lagged Analysis During the Post-COVID-19 Transition

**DOI:** 10.7759/cureus.107845

**Published:** 2026-04-27

**Authors:** Yoshinobu Fujita, Hiromu Nagaura, Yuko Taniya, Atsuhiro Satsuma, Toshihiko Tsutsumi, Tetsuro Noda

**Affiliations:** 1 Faculty of Psychology, Osaka University of Human Sciences, Settsu, JPN; 2 Graduate School of Education, Hokkaido University of Education, Asahikawa, JPN; 3 Department of Psychiatry, Higashi Fuse Noda Clinic, Higashiosaka, JPN; 4 Faculty of Modern Social Studies, Otemae University, Osaka, JPN; 5 Department of Psychiatry, Sekizen Hospital, Tsuyama, JPN

**Keywords:** covid-19, cross-lagged analysis, k6 scale, loneliness, longitudinal study, media consumption, psychological distress, university students

## Abstract

Introduction

The COVID-19 pandemic significantly affected university students' mental health, and loneliness is frequently cited as a key contributor to psychological distress. However, whether loneliness drives distress or distress generates loneliness remains unresolved. This study examined this question during the post-COVID-19 transition, when social opportunities had been restored, but psychological effects may have persisted.

Methods

A longitudinal web-based survey was conducted with Japanese university students at two time points: October 2022 (T1, during COVID-19 restrictions) and February 2024 (T2, post-restrictions). From 3,000 respondents at T1 and 3,075 at T2, 230 (7.7%; 95 male respondents, 135 female respondents; mean age = 20.3 years) completed both surveys. Cross-lagged regression analysis was used to examine the bidirectional relationships between the Kessler Psychological Distress Scale (K6) and UCLA Loneliness Scale (three-item version) scores.

Results

Loneliness decreased significantly from T1 to T2 (*p* = 0.017, *d* = 0.16), whereas distress did not. K6 scores at T1 predicted loneliness at T2 (β = 0.16, 95% confidence interval (CI) = 0.04, 0.28, *p* = 0.011), but loneliness at T1 did not predict K6 scores at T2 (β = 0.05, 95% CI = -0.06, 0.16, *p* = 0.333). An equality constraint test indicated that the difference between the two cross-lagged paths was not statistically significant (Δχ²(1) = 1.26, *p* = 0.263); therefore, the asymmetric pattern is suggestive of temporal precedence rather than confirmatory evidence of a unidirectional effect. T1 distress also predicted increased passive media consumption at T2; however, T1 behavioral changes did not predict T2 psychological outcomes, except that exercise was associated with lower subsequent distress.

Conclusions

These findings provide preliminary evidence that, over the 16-month period from October 2022 to February 2024, psychological distress is temporally associated with subsequent loneliness rather than the reverse. In this context, loneliness may partly represent a cognitive manifestation of underlying distress. Interventions for lonely university students would benefit from addressing underlying psychological distress, rather than focusing solely on increasing social connections.

## Introduction

COVID-19 and university students' mental health

Psychological distress refers to a state of non-specific emotional suffering characterized by symptoms of depression and anxiety that arise in response to stressors and are not attributable to a specific psychiatric disorder [1]. The COVID-19 pandemic has profoundly affected the mental health of university students worldwide [2]. Social distancing, campus closures, and the abrupt shift to online learning disrupted the developmental experiences typical of young adulthood, such as identity exploration, relationship formation, and growing autonomy. In Japan, elevated anxiety and depression have been documented among university students using the Kessler Psychological Distress Scale (K6) [3]. Importantly, mental health deterioration persisted even after restrictions were eased, suggesting that the psychological impact extended beyond the acute crisis [4].

The policy environment shifted markedly between the two study time points. At T1 (October 2022), Japan still maintained widespread mask-wearing recommendations and restrictions on large gatherings. By early 2023, mask mandates in indoor public spaces were relaxed and left to individual discretion. In May 2023, COVID-19 was reclassified in Japan from a Category II to a Category V infectious disease under the Infectious Diseases Control Law, which removed most legal restrictions on daily activities. Universities resumed in-person classes and extracurricular activities during the 2023 academic year, and by T2 (February 2024), campus life had largely returned to pre-pandemic patterns. Throughout this manuscript, we use the term "post-COVID-19 transition" to refer specifically to this 16-month interval (October 2022 to February 2024), during which Japan progressively moved from active pandemic restrictions to the functional restoration of pre-pandemic campus life.

Loneliness emerged as a particularly prominent concern. University students faced unprecedented social isolation during campus closures and activity restrictions [5]. Multiple studies have reported associations between loneliness and psychological distress, leading to the widespread assumption that pandemic-induced loneliness was a primary driver of mental health deterioration [6].

Loneliness: conceptualization and measurement

Examining the direction of this relationship requires careful attention to what loneliness actually measures. Loneliness is commonly defined as the subjective experience of perceived deficiencies in one's social relationships, whether in terms of quantity or quality [7]. It is not simply the absence of social contact but a felt discrepancy between desired and actual connections. The UCLA Loneliness Scale [8], one of the most widely used measures, assesses subjective perceptions rather than objective indicators of isolation.

This distinction is important. Being alone and feeling lonely are not the same. Objective isolation, having few social contacts or network ties, correlates moderately with subjective loneliness [9]. Some individuals feel lonely despite having extensive networks, while others with limited contacts do not [10]. Thus, loneliness is not merely a reflection of one's social circumstances. It is shaped by cognitive appraisal.

This raises an important question about the extent to which loneliness reflects an accurate perception of one's social environment, rather than a bias colored by one's psychological state. Cognitive theories of depression hold that depressed individuals exhibit negative biases in perceiving, remembering, and interpreting social information [11]. If loneliness is partly a cognitive appraisal, it may be susceptible to such biases. Psychological distress can lead individuals to perceive themselves as lonelier than their objective circumstances warrant.

The directionality debate

The predominant view is that loneliness is a precursor to depression and anxiety [12]. Evolutionary accounts suggest that loneliness functions as an aversive signal motivating social reconnection and that chronic loneliness triggers physiological stress responses that increase vulnerability to mental health problems [13]. Longitudinal studies have supported this pathway [14].

However, the causal arrow may point in the opposite direction or at least be bidirectional. Depression is characterized by social withdrawal, reduced motivation for social engagement, and negative interpretation of social interactions, features well documented in clinical accounts of depression [11]. These features could lead depressed individuals to reduce their social activities and perceive existing relationships more negatively, thereby increasing their loneliness. A recent cross-lagged analysis of European adults found that depression predicted loneliness in females, but not vice versa [15].

Among university students, the evidence is inconclusive. Some longitudinal studies support the loneliness-to-depression pathway, while others have found bidirectional effects or stronger evidence of depression predicting loneliness [16]. A cross-lagged panel network analysis of Chinese college students found that loneliness predicted increased depressive symptoms during the acute pandemic period [17]. Whether these patterns persist in the post-COVID-19 transition, when social restrictions have been lifted, but psychological effects may linger, remains unclear.

This directionality question also extends to behavioral consequences. During the pandemic, behaviors such as Internet use, social media engagement, and video streaming increased among university students [18]. The Interaction of Person-Affect-Cognition-Execution (I-PACE) model provides a framework for understanding how negative affective states can lead to problematic media use [19,20]. According to this model, individuals with specific predisposing characteristics (Person) experience negative affect (Affect), which interacts with cognitive biases and expectancies (Cognition) and reduced executive control (Execution) to drive habitual engagement in mood-regulating behaviors such as excessive Internet, social media, and video streaming use. Under this framework, media consumption is conceptualized as a downstream consequence of negative affect rather than its cause, at least in the short to medium term. Testing whether distress precedes behavioral changes or vice versa can help to clarify this issue.

The present study

This study examined the temporal relationships between psychological distress and loneliness among Japanese university students over a 16-month period spanning the post-COVID-19 transition. Framed as tests of temporal precedence rather than causation, two hypotheses were examined: (1) psychological distress at Time 1 would show a stronger predictive association with loneliness at Time 2 than the reverse pathway, and (2) consistent with the I-PACE model, distress at Time 1 would predict increased media consumption at Time 2, whereas behavioral changes at Time 1 would not predict subsequent psychological outcomes.

## Materials and methods

Study design

This was a longitudinal study using a two-wave web-based survey with a 16-month interval. Data were collected at Time 1 (T1) in October 2022 and Time 2 (T2) in February 2024. The interval between waves was determined by a practical constraint rather than a theoretical rationale: T1 respondents who entered university in 2020, the first year of the pandemic, would graduate by March 2024 under the standard four-year program, so the follow-up survey was scheduled within this window. At T1, Japan was still under various COVID-19 restrictions that affected university activities. By T2, most restrictions had been lifted, and campus life had largely returned to normal. The primary aim was to examine bidirectional relationships between psychological distress and loneliness using cross-lagged regression analysis.

Participants and eligibility criteria

Participants were recruited through convenience sampling via the registered online panel of a commercial survey company (Macromill, Inc., Tokyo, Japan). At each wave, invitations were issued to panel members who had registered themselves as currently enrolled university or graduate school students in Japan. Eligibility criteria for participation at each wave were as follows: (a) current enrollment as a university or graduate school student in Japan, (b) age 18 years or older, and (c) completion of the survey within the fielding window.

Of the total respondents at each wave (T1: n = 3,000; T2: n = 3,075), the commercial panel operator (Macromill, Inc.) identified respondents who had participated in both waves using internal respondent identifiers assigned at T1 and provided the research team with a linked dataset matched at the individual level. This procedure yielded 233 respondents with records at both time points. Three participants who did not provide binary sex information (male/female) at T2 were excluded to enable sex-stratified analyses, resulting in a final analytic sample of 230 (95 males and 135 females; 7.7% of T1 respondents and 7.5% of T2 respondents). All 230 participants had complete responses on the primary outcome measures (K6 and UCLA Loneliness Scale). The mean age at T1 was 20.3 years (standard deviation (SD) = 1.1, range = 18-23).

The relatively low cross-wave overlap reflects the commercial panel's design: respondents at each time point were drawn independently from the company's registered pool, rather than through a pre-registered cohort design. Sample size adequacy for the cross-lagged regression analysis is addressed in the Statistical analysis subsection below. Potential selection biases arising from this procedure are discussed in the Limitations section.

Data collection

Data were collected electronically via the commercial survey company's online platform at both time points. The survey was self-administered, and participants accessed it through the company's registered panel system. Respondents received standard panel incentive points from the survey company for participation. No personally identifying information beyond demographic variables was collected by the research team. The cross-wave linkage procedure conducted by the panel operator is described in the Participants and eligibility criteria subsection above.

Measures

Psychological Distress

Psychological distress was assessed using the Kessler Psychological Distress Scale (K6) [21], a six-item measure of anxiety and depressive symptoms experienced during the past 30 days. Items are rated from 0 (none of the time) to 4 (all of the time), and total scores range from 0 to 24, with higher scores indicating greater distress. Established cut-points categorize scores of 5-12 as indicating moderate distress and scores of 13 or higher as indicating severe distress likely to reflect a probable mental disorder [21]. The Japanese version has demonstrated good reliability and validity [22]. In the present sample, Cronbach's α was 0.89 at T1 and 0.91 at T2.

Loneliness

Loneliness was assessed with the three-item UCLA Loneliness Scale [23], a brief version of the original scale [8]. Three items are rated from 1 (hardly ever) to 4 (often), and total scores range from 3 to 12, with higher scores indicating greater loneliness. Unlike the K6, no universally agreed-upon clinical cut-points exist for the three-item UCLA Loneliness Scale; researchers have proposed various thresholds (e.g., scores ≥ 6 as "lonely" in some studies), but these are not consensus standards [23]. Scores were therefore analyzed as a continuous variable. In the present sample, Cronbach's α was 0.85 at T1 and 0.87 at T2.

Social support at T2 was assessed using the Lubben Social Network Scale-6 (LSNS-6) [24], which comprises three items on family networks and three on friend networks. Each item asks about the number of people with whom the respondent has a specific type of social contact (e.g., "How many relatives do you see or hear from at least once a month?"), and is rated on a six-point scale: 0 ("none"), 1 ("one"), 2 ("two"), 3 ("three or four"), 4 ("five to eight"), and 5 ("nine or more"). The total score ranges from 0 to 30, with higher scores indicating larger perceived social networks. A cut-point of <12 has been proposed to identify individuals at risk for social isolation [24]. The LSNS-6 has demonstrated good internal consistency in prior validation studies [24]. The LSNS-6 was administered only at T2 and was used in the robustness check to examine whether the cross-lagged findings were independent of social support availability.

Behavioral Changes

Participants reported whether each of the following six behaviors had changed since before the pandemic: use of social networking services (SNS), YouTube viewing, video streaming, gaming, exercise, and alcohol consumption. Response options were "increased," "unchanged," "decreased," or "not applicable"; the last option was provided for behaviors the respondent did not engage in. Those who selected "not applicable" were excluded from the relevant analysis. For correlation analyses, responses were coded as -1 (decreased), 0 (unchanged), or 1 (increased). These items were constructed for the present longitudinal research program and have not undergone formal psychometric validation, such as a dedicated pilot study or expert review. Their face-valid interpretation as indicators of directional behavioral change is acknowledged as a limitation.

Statistical analysis

All analyses were performed using the Statistical Package for the Social Sciences (SPSS) version 27 (IBM Corp., Armonk, NY). The significance level was set at p < 0.05 (two-tailed). Missing data on the primary outcome measures were nil: the commercial panel survey platform required completion of the K6 and UCLA Loneliness Scale items before respondents could proceed through the questionnaire, so no imputation was needed. Behavioral change items with "not applicable" responses were listwise-excluded from the relevant correlation only, with the remaining sample sizes reported for each analysis.

Paired t-tests were used to examine changes in K6 and UCLA loneliness scores from T1 to T2, with Cohen's d as the effect size. Although the distributions of these total scores showed some deviation from normality, paired t-tests are known to be robust to non-normality at the present sample size (N = 230), which exceeds the thresholds at which the central limit theorem ensures adequate control of type I error rates. As a sensitivity check, Wilcoxon signed-rank tests were conducted in parallel for all paired comparisons; the results are reported alongside the t-test results.

Cross-lagged regression analysis was conducted to examine the bidirectional relationships between K6 and UCLA loneliness scores across the two time points. All variables were z-standardized, and ordinary least squares regression was used to estimate the autoregressive and cross-lagged paths. The resulting two-variable, two-wave cross-lagged model is saturated (df = 0); by definition, χ² = 0, comparative fit index (CFI) = 1.00, and root mean square error of approximation (RMSEA) = 0.00, so conventional incremental fit indices are uninformative for this model. An equality constraint test compared the freely estimated model with a model constraining the two cross-lagged paths to be equal using a likelihood ratio test (Δχ²). The analytic sample of N = 230 exceeded the commonly recommended minimum of 200 cases for stable parameter estimates in cross-lagged regression with two primary variables [25]. A post hoc sensitivity analysis indicated that N = 230 provided 80% power (α = 0.05, two-tailed) to detect a standardized cross-lagged coefficient of |β| ≥ 0.15 (*f*² = 0.034), and that the observed effect of β = 0.16 (f² = 0.029) was detected with 73% power. For the equality constraint test, the estimated post hoc power was approximately 20%, and approximately >1,430 participants would be required to achieve 80% power for the observed difference between the two cross-lagged coefficients.

As a robustness check on the cross-lagged findings, two hierarchical regression analyses were conducted to test whether each cross-lagged effect remained after adjusting for social support at T2. For each outcome (T2 loneliness, T2 K6), Step 1 entered the corresponding T1 autoregressive predictor and T2 LSNS-6, and Step 2 added the cross-lagged predictor. The change in R² was tested with an F change test.

For associations involving the ordinally coded behavioral change variables (-1, 0, 1), Spearman rank-order correlation coefficients (r_s_) were used, which do not assume interval-level measurement. These correlations were computed for (a) T1 psychological variables with T2 behavioral changes and (b) T1 behavioral changes with T2 psychological outcomes.

Ethical considerations

Prior to the survey, the participants were presented with a written explanation of the study's purpose, procedures, and data handling. Only those who confirmed their understanding and clicked the consent button proceeded to the main survey section. The study was conducted in accordance with the Declaration of Helsinki and approved by the Ethics Committee of the Osaka University of Human Sciences (reference number: 2022-5).

## Results

Descriptive statistics

The analytical sample comprised 230 Japanese university students (95 male students and 135 female students; mean age at T1 = 20.3 years, SD = 1.1, range = 18-23). The academic year distribution at T1 was as follows: Year 1 (n = 35), Year 2 (n = 68), Year 3 (n = 108), and Year 4 (n = 19). Table 1 presents the descriptive statistics and within-person comparisons between T1 and T2 for the main study variables, reported separately for the overall sample and by sex.

**Table 1:** Descriptive Statistics and Paired-Samples Comparisons Between T1 and T2 (N = 230) K6: range = 0-24; α = 0.89 at T1, 0.91 at T2 UCLA (3-item version): range = 3-12; α = 0.85 at T1, 0.87 at T2 LSNS-6: range = 0-30 T1 = October 2022; T2 = February 2024 Mean diff = T1 - T2 K6: Kessler Psychological Distress Scale, UCLA: UCLA Loneliness Scale, LSNS-6: Lubben Social Network Scale-6, CI: confidence interval, d: Cohen's d for paired samples, Wilcoxon p: p-value from the Wilcoxon signed-rank test conducted as a sensitivity check given non-normal score distributions, M: mean, SD: standard deviation

Variable	Group	Number	T1 (M (SD))	T2 (M (SD))	Mean diff	95% CI	t	df	p	d	Wilcoxon p
K6 distress	Overall	230	6.30 (6.21)	5.83 (6.38)	0.47	-0.17, 1.11	1.46	229	0.147	0.10	0.119
K6 distress	Males	95	5.86 (5.77)	4.75 (5.57)	1.12	0.02, 2.21	2.02	94	0.047	0.21	0.054
K6 distress	Females	135	6.61 (6.51)	6.59 (6.80)	0.02	-0.75, 0.78	0.04	134	0.969	0.00	0.729
UCLA loneliness	Overall	230	7.37 (2.64)	7.00 (2.64)	0.37	0.07, 0.67	2.39	229	0.017	0.16	0.031
UCLA loneliness	Males	95	7.16 (2.54)	6.62 (2.50)	0.54	0.01, 1.07	2.01	94	0.047	0.21	0.056
UCLA loneliness	Females	135	7.52 (2.71)	7.27 (2.72)	0.25	-0.11, 0.62	1.37	134	0.174	0.12	0.285
LSNS-6 (T2 only)	Overall	230	-	16.81 (5.99)	-	-	-	-	-	-	-
LSNS-6 (T2 only)	Males	95	-	17.20 (6.35)	-	-	-	-	-	-	-
LSNS-6 (T2 only)	Females	135	-	16.54 (5.74)	-	-	-	-	-	-	-

At the overall level, K6 scores did not change significantly from T1 (M = 6.30, SD = 6.21) to T2 (M = 5.83, SD = 6.38) (paired t-test: t(229) = 1.46, p = 0.147, d = 0.10, 95% CI = -0.17, 1.11). The accompanying Wilcoxon signed-rank test yielded the same conclusion (p = 0.119). However, the pattern differed by sex: males showed a significant decrease in K6 (t(94) = 2.02, p = 0.047, d = 0.21, 95% CI = 0.02, 2.21; Wilcoxon p = 0.054), whereas females showed no change (t(134) = 0.04, p = 0.969, d = 0.00).

Loneliness scores decreased significantly at the overall level from T1 (M = 7.37, SD = 2.64) to T2 (M = 7.00, SD = 2.64) (t(229) = 2.39, p = 0.017, d = 0.16, 95% CI = 0.07, 0.67; Wilcoxon p = 0.031). By sex, male participants again showed a significant decrease (t(94) = 2.01, p = 0.047, d = 0.21), whereas the change in female participants did not reach significance (t(134) = 1.37, p = 0.174, d = 0.12). The mean LSNS-6 score at T2 was 16.81 (SD = 5.99, range = 6-31), indicating that the sample, on average, had social networks above the social-isolation cut-point of 12 [24].

Bivariate Pearson correlations among the primary study variables were in expected directions and magnitudes. Within-construct correlations across time were substantial (K6 T1 × K6 T2: r = 0.70; loneliness T1 × loneliness T2: r = 0.61; both p < 0.001), reflecting moderate-to-strong rank-order stability. Cross-construct, cross-time correlations were also significant: K6 T1 × loneliness T2 (r = 0.43, p < 0.001) and loneliness T1 × K6 T2 (r = 0.41, p < 0.001). The T2 LSNS-6 showed a small negative correlation with T2 K6 (r = -0.16, p = 0.019) and a non-significant correlation with T2 loneliness (r = -0.13, p = 0.053).

Cross-lagged analysis of psychological distress and loneliness

**Figure 1:**
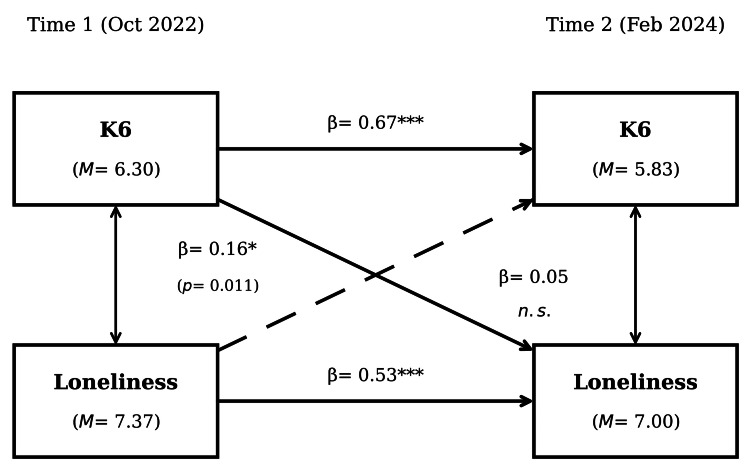
Cross-Lagged Model of Psychological Distress (K6) and Loneliness (N = 230) Standardized coefficients are presented. Autoregressive paths are shown horizontally, and cross-lagged paths are shown diagonally. Solid lines indicate significant paths; dashed lines indicate non-significant paths. *p < 0.05, ***p < 0.001 K6: Kessler Psychological Distress Scale

Figure 1 and Table 2 present the cross-lagged regression results. Both variables showed significant autoregressive stability (K6: β = 0.67, 95% CI = 0.56, 0.78, p < 0.001; loneliness: β = 0.53, 95% CI = 0.41, 0.65,p < 0.001), indicating moderate-to-strong continuity in individual differences over time.

**Table 2:** Cross-Lagged Regression Paths Between Psychological Distress (K6) and Loneliness (N = 230) Standardized regression coefficients (β) are presented. All variables were z-standardized prior to analysis. The cross-lagged model was saturated (df = 0); by definition, χ² = 0, CFI = 1.00, RMSEA = 0.00. The equality constraint test compared the freely estimated model with one constraining the two cross-lagged paths to be equal, using a likelihood ratio test (Δχ²). CFI: comparative fit index, RMSEA: root mean square error of approximation, T1: Time 1 (October 2022), T2: Time 2 (February 2024)

Path	β	SE	95% CI	t	p
K6 T1 → K6 T2 (autoregressive)	0.67	0.056	0.56, 0.78	12.03	<0.001
Loneliness T1 → Loneliness T2 (autoregressive)	0.53	0.061	0.41, 0.65	8.59	<0.001
K6 T1 → Loneliness T2 (cross-lagged)	0.16	0.061	0.04, 0.28	2.55	0.011
Loneliness T1 → K6 T2 (cross-lagged)	0.05	0.056	-0.06, 0.16	0.97	0.333
Equality constraint test	Δχ²(1) = 1.26				0.263

The cross-lagged paths were asymmetric. K6 at T1 significantly predicted loneliness at T2, after controlling for T1 loneliness (β = 0.16, 95% CI = 0.04, 0.28, p = 0.011). In the reverse direction, loneliness at T1 did not predict K6 at T2 when controlling for T1 K6 (β = 0.05, 95% CI = -0.06, 0.16, p = 0.333). This pattern is consistent with distress temporally preceding increases in loneliness, but not vice versa.

An equality constraint test, however, indicated that the difference between the two cross-lagged paths was not statistically significant (Δχ²(1) = 1.26, p = 0.263). Although one path reached statistical significance and the other did not, their magnitudes were not significantly different from one another. This observation has an important interpretative implication: the data are consistent with asymmetric temporal precedence (distress → loneliness being stronger than the reverse), but the present study had insufficient statistical power to confirm that this asymmetry itself is statistically robust. Accordingly, the asymmetric pattern should be regarded as suggestive rather than conclusive.

Robustness check: adjusting for social support at T2

To examine whether the cross-lagged pattern could be accounted for by deficits in social support, two hierarchical regressions were conducted, one for each cross-lagged path (Table 3). For T2 loneliness as the outcome, T1 loneliness and T2 social support (LSNS-6) were entered in Step 1, and T1 K6 was added in Step 2. T2 social support did not significantly predict T2 loneliness (β = -0.05, p = 0.310), whereas T1 K6 remained a significant predictor even after controlling for T1 loneliness and T2 social support (β = 0.15, p = 0.015, ΔR² = 0.016). For T2 K6 as the outcome, T1 loneliness did not predict T2 K6 after controlling for T1 K6 and T2 social support (β = 0.05, p = 0.366, ΔR² = 0.002). Thus, the asymmetric cross-lagged pattern was not attributable to differences in social support at T2.

**Table 3:** Hierarchical Regression Analyses Examining Whether the Cross-Lagged Effects Are Robust After Adjusting for Social Support at T2 (N = 230) Standardized regression coefficients (β) are reported. All predictors were z-standardized prior to analysis. The analysis tests whether the cross-lagged effects (T1 K6 → T2 Loneliness and T1 Loneliness → T2 K6) remain after social support at T2 (LSNS-6) is included in the model. K6: Kessler Psychological Distress Scale, LSNS-6: Lubben Social Network Scale-6, T1: Time 1 (October 2022), T2: Time 2 (February 2024)

Outcome	Step	Predictor	β	SE	p	R²	ΔR²	F change (1, 226)	p (F change)
T2 Loneliness	Step 1	T1 Loneliness	0.60	0.053	<0.001	0.373	-	-	-
		T2 LSNS-6	-0.06	0.053	0.226				
	Step 2	T1 Loneliness	0.52	0.061	<0.001	0.389	0.016	6.05	0.015
		T2 LSNS-6	-0.05	0.052	0.310				
		T1 K6	0.15	0.061	0.015				
T2 K6 distress	Step 1	T1 K6	0.69	0.048	<0.001	0.493	-	-	-
		T2 LSNS-6	-0.07	0.048	0.156				
	Step 2	T1 K6	0.66	0.056	<0.001	0.495	0.002	0.82	0.366
		T2 LSNS-6	-0.07	0.048	0.170				
		T1 Loneliness	0.05	0.056	0.366				

Psychological distress and subsequent behavioral changes

Table 4 presents the Spearman correlations between T1 psychological variables and T2 behavioral changes. T1 K6 was associated with increased YouTube viewing (r_s_ = 0.18, p = 0.006), increased video streaming (r_s_ = 0.16, p = 0.029), and decreased exercise (r_s_ = -0.16, p = 0.036) at T2, respectively. T1 loneliness was associated with increased SNS use (r_s_ = 0.17, p = 0.013) and video streaming (r_s_ = 0.23, p = 0.002). Neither variable showed significant associations with changes in either gaming or alcohol consumption.

**Table 4:** Spearman Correlations Between T1 Psychological Variables and T2 Behavioral Changes Behavioral change was coded as -1 (decreased), 0 (unchanged), 1 (increased). "Excluded" refers to participants who selected "not applicable" or did not respond; these were excluded from the relevant correlation. *p < 0.05, **p < 0.01 T1: Time 1 (October 2022), T2: Time 2 (February 2024), SNS: social networking services

T2 behavioral change	Number	Excluded	Increased	Unchanged	Decreased	K6 (T1) r_s_	Loneliness (T1) r_s_
SNS use	215	15	83	116	16	0.12	0.17*
YouTube viewing	221	9	105	100	16	0.18**	0.12
Video streaming	179	51	72	91	16	0.16*	0.23**
Gaming	153	77	37	90	26	0.01	0.14
Exercise	178	52	30	109	39	-0.16*	-0.08
Alcohol consumption	146	84	31	101	14	-0.12	-0.06

T1 behavioral changes did not predict T2 psychological outcomes

Table 5 presents the Spearman correlations between T1 behavioral changes and T2 psychological outcomes. No significant associations emerged between T2 K6, T2 loneliness, and change scores for either variable.

**Table 5:** Spearman Correlations Between T1 Behavioral Changes and T2 Psychological Outcomes and Change Scores Behavioral change was coded as -1 (decreased), 0 (unchanged), 1 (increased). "Excluded" refers to participants who selected "not applicable" or did not respond. *p < 0.05 Chg: change score (T2 minus T1), T1: Time 1, T2: Time 2, SNS: social networking services

T1 behavioral change	Number	Excluded	Increased	Unchanged	Decreased	K6 (T2) r_s_	Loneliness (T2) r_s_	K6 Chg r_s_	Loneliness Chg r_s_
SNS use	221	9	111	99	11	-0.02	0.13	0.00	0.05
YouTube viewing	219	11	133	77	9	-0.01	0.07	0.05	-0.11
Video streaming	158	72	88	60	10	-0.10	0.00	-0.02	-0.12
Gaming	158	72	60	73	25	-0.10	0.06	0.00	0.00
Exercise	190	40	55	94	41	-0.16*	-0.04	-0.05	0.08
Alcohol consumption	101	129	35	58	8	-0.06	0.12	-0.14	-0.06

The sole exception was exercise. Increased exercise at T1 was associated with a lower K6 score at T2 (r_s_ = -0.15, p = 0.032), but not with loneliness at T2 (r_s_ = -0.04, p = 0.611). This asymmetric pattern, in which T1 psychological variables were associated with T2 behavioral changes, but T1 behavioral changes were not associated with T2 psychological outcomes, is consistent with behavioral changes during this period following from distress rather than independently contributing to it.

## Discussion

This study provides the first longitudinal test of the distress-to-loneliness direction among post-COVID-19 Japanese university students. The cross-lagged analysis reveals an asymmetry at the point-estimate level (distress predicts subsequent loneliness, but not vice versa), although the equality constraint test lacked sufficient power to confirm that the two paths differ statistically. Together, these findings suggest that loneliness in this population may function partly as a cognitive symptom of underlying distress rather than as a reflection of objective social deficits.

The finding that distress predicted subsequent loneliness, but not vice versa, challenges the common assumption that loneliness is primarily a cause of mental health problems. Our results align with those of a recent European cross-lagged study in which depression predicted loneliness, but not vice versa [15]. However, they contrast with acute pandemic studies in which loneliness was a stronger predictor of depressive symptoms [17].

We interpret this discrepancy as reflecting contextual differences between the two periods. During enforced social isolation, when campus closures directly limited interpersonal contact, loneliness may have driven distress because individuals were objectively prevented from meeting their social needs. During the post-COVID-19 transition examined here, restrictions were lifted, and social opportunities largely returned to normal. Under these circumstances, loneliness may shift from reflecting objective social deprivation to a more subjective, cognitively mediated experience.

This interpretation is consistent with Beck's cognitive theory of depression [11], which holds that depressed individuals exhibit systematic negative biases in perceiving, interpreting, and remembering social information. Following Beck's formulation, such biases could lead distressed individuals to perceive themselves as lonelier than their circumstances warrant, experiencing loneliness as a cognitive symptom of distress rather than as a reflection of actual social deficits.

The robustness check supports this interpretation (Table 3). At T2, social support (LSNS-6) did not predict loneliness after controlling for distress, whereas the effect of T1 distress on T2 loneliness remained significant after adjusting for T2 social support. Students who experienced greater distress at T1 reported higher loneliness at T2, regardless of their social network size. We interpret this pattern as consistent with the view that loneliness in this context functions as a cognitive appraisal shaped by distress rather than a reflection of objective social deficits.

Sex differences in psychological recovery

Changes over time also differed by sex (Table 1). Men showed significant decreases in both K6 distress and loneliness (both p = 0.047, d = 0.21), whereas women showed essentially no change in either measure. The recovery observed at the sample level was therefore largely driven by men, continuing a pattern previously reported in this population [3]. A plausible account centers on how female students typically regulate affect through close, face-to-face peer relationships; pandemic restrictions disrupted this channel, and although in-person opportunities were restored by T2, relationships that lapsed during the acute phase are not necessarily rebuilt by the mere availability of contact. The additional burden of reactivating peer networks may have left female students with less affect-regulatory benefit from the return of social opportunity than their male peers received. Because measurement invariance by sex was not formally examined, sex-stratified results should be regarded as descriptive and hypothesis-generating.

Behavioral changes following from distress

The pattern in which distress was associated with subsequent behavioral changes, but not vice versa, is consistent with the I-PACE model [19,20]. Students with higher T1 distress were more likely to report increased YouTube viewing and video streaming at T2. Those with higher T1 loneliness reported increased SNS use and video streaming usage. These findings are consistent with the possibility that students turned to media consumption to cope with negative emotional states.

The reverse pathway was largely absent in this study. This asymmetry is more consistent with media use following from distress than with the reverse temporal direction during this period. We cannot rule out the possibility that chronic excessive use contributes to psychological problems over longer timeframes; however, the present data speak to the short-to-medium-term direction of association, which runs from distress to behavior.

Exercise was the only exception. Increased exercise at T1 was associated with lower distress at T2, consistent with the well-documented mental health benefits of physical activity [26]. Unlike passive media consumption, exercise may actively contribute to psychological well-being.

Clinical and practical implications

If loneliness among university students often reflects underlying distress rather than objective social isolation, interventions focused solely on increasing social connections may be insufficient. A student reporting high loneliness may benefit from assessment and intervention for depression or anxiety symptoms that may be shaping their perception of the social world, rather than from social opportunities alone. This implication is consistent with recent preliminary evidence that modular cognitive-behavioral interventions addressing both maladaptive social cognitions and co-occurring emotional difficulties can produce substantial reductions in loneliness alongside improvements in anxiety and depression in young people [27].

In the university context, student counseling services that already offer cognitive-behavioral approaches for depression and anxiety may be well positioned to monitor changes in loneliness as a secondary outcome and to incorporate cognitive restructuring of distorted social appraisals into existing distress-focused protocols. Routine screening with brief instruments such as the K6, combined with an assessment of perceived loneliness, could help identify students whose loneliness is driven by psychological distress rather than by a lack of social opportunity, allowing interventions to be appropriately targeted.

Limitations and future directions

This study has several limitations. First, the analytic sample of 230 participants was derived from post hoc matching across two independently drawn commercial panel waves (T1: n = 3,000, T2: n = 3,075), yielding cross-wave overlap rates of 7.7% and 7.5%, respectively. This procedure may over-represent respondents with stable panel membership, and selection bias cannot be ruled out. Because the panel operator conducted the cross-wave linkage using proprietary internal identifiers and provided only the matched dataset, individual-level T1 data for non-matched respondents were unavailable, precluding a formal attrition analysis comparing completers with non-completers. As all outcomes were also self-reported within a single survey platform, common-method variance could inflate cross-construct associations, although the consistency of the asymmetric pattern across psychological and behavioral outcomes makes method variance an unlikely sole explanation.

Second, the equality constraint test did not detect a statistically significant difference between the two cross-lagged paths (Δχ²(1) = 1.26, p = 0.263). Post hoc power for this test was approximately 20%, and an estimated 1,430 participants would be needed to achieve 80% power for the observed difference. Larger samples will be needed to determine whether the two paths genuinely differ in magnitude.

Third, cross-lagged regression with two waves can provide evidence consistent with temporal precedence but cannot establish causation, and between-person and within-person effects cannot be separated in this design. Loneliness was measured with the three-item UCLA Loneliness Scale [23], which assesses a narrower content domain than the full 20-item version [8]; replication with the full scale would provide more stable estimates and broader coverage of the loneliness construct. Designs with three or more waves would enable random-intercept cross-lagged panel modeling [28] to separate between- from within-person processes, and intensive longitudinal methods such as ecological momentary assessment could clarify the short-term dynamics linking distress and loneliness. Cross-cultural replication and intervention studies targeting depressive cognitions in lonely students would further test the generalizability and practical implications of the present findings.

## Conclusions

This study provides preliminary longitudinal evidence consistent with the hypothesis that psychological distress is temporally associated with subsequent loneliness among Japanese university students during the post-COVID-19 transition. The asymmetric recovery of these constructs (loneliness decreased with restored social contact, while distress remained stable) and the persistence of the distress-to-loneliness path after adjustment for social support together suggest that loneliness in this context may partly function as a cognitive manifestation of underlying distress. The equality constraint test did not confirm a statistically significant difference between the two cross-lagged paths, however, so the evidence should be read as suggestive rather than definitive. Consistent with the I-PACE framework, passive media consumption at T2 was predicted by T1 distress but did not predict subsequent psychological outcomes, whereas exercise showed a protective pattern. Interventions for lonely university students may benefit from addressing underlying distress in addition to, rather than instead of, efforts to expand social contact. Research with larger samples, more frequent assessments, and diverse cultural contexts will help clarify the potentially context-dependent relationship between loneliness and psychological distress.
